# Epigallocatechin gallate enhances sympathetic heart rate variability and decreases blood pressure in obese subjects: a randomized control trial

**DOI:** 10.1038/s41598-024-72269-3

**Published:** 2024-09-16

**Authors:** Kittikorn Tommy Wilasrusmee, Chantacha Sitticharoon, Issarawan Keadkraichaiwat, Pailin Maikaew, Kitchaya Pongwattanapakin, Saimai Chatree, Rungnapa Sririwichitchai, Malika Churintaraphan

**Affiliations:** Department of Physiology, Faculty of Medicine Siriraj Hospital, Mahidol University, 2 Wanglang Rd., Siriraj, Bangkoknoi, Bangkok, 10700 Thailand

**Keywords:** Obesity, EGCG, HRV, MAP, Metabolic, Hormones, Cardiovascular biology, Metabolism, Physiology, Endocrinology

## Abstract

This study aimed to investigate effects of epigallocatechin gallate (EGCG) on blood pressure (BP) and autonomic nervous system, indicated by 5-min heart rate variability (HRV) measurement in obese subjects, and determine correlations of BP with metabolic factors. In a double-blind, randomized controlled trial, obese subjects (n = 30) were randomly allocated to receive 150 mg EGCG (n = 15) or placebo (n = 15) twice a day without dietary restrictions. After 8-week EGCG treatment, systolic blood pressure (SBP), diastolic blood pressure (DBP), and mean arterial pressure (MAP) significantly decreased, while the low-frequency (LF) to high-frequency power (HF) ratio (LF/HF ratio) significantly increased (P < 0.05 all), indicating a shift toward sympathetic dominance, either directly or indirectly after BP lowering. SBP had positive correlations with obesity parameters, leptin, insulin, and insulin resistance but had a negative correlation with insulin sensitivity. DBP was positively correlated with age and HF in normalized unit, but negatively correlated with height and LF in ms^2^. High-density lipoprotein cholesterol (HDL-C) was negatively correlated with SBP, DBP, and MAP reflecting its protective effect against elevated BP. In conclusion, the 8-week EGCG treatment decreased BP and increased the LF/HF ratio, reflecting increased sympathetic activity, either a direct EGCG effect or an indirect compensatory response following BP reduction.

## Introduction

Green tea, derived from *Camellia sinensis,* is globally popular, containing approximately 37–56% flavonoids, with predominant catechins such as epicatechin (EC), epicatechin-3-gallate (ECG), epigallocatechin (EGC) and epigallocatechin-3-gallate (EGCG)^1,2^. EGCG, known for its potent antioxidant and prooxidant properties, has potential preventive effects against diseases like cancer and cardiovascular diseases^1–3^.

EGCG has been shown to reduce systolic blood pressure (SBP) and diastolic blood pressure (DBP) in obese patients^1,4–6^, which is beneficial for decreasing the risk of metabolic syndrome and its complications^7^. The effects of EGCG on blood pressure (BP) reduction are mediated through several mechanisms. EGCG has a vasodilatory effect by stimulating nitric oxide (NO) and inhibiting endothelin 1 (ET-1) production^8^. Furthermore, in Angiotensin-II induced hypertensive mice, EGCG demonstrated antioxidative properties leading to NO production and vasodilation^9^.

In relation to these cardiovascular effects, the autonomic nervous system (ANS), comprising both sympathetic and parasympathetic branches, plays a crucial role in maintaining cardiovascular, renal, and metabolic functions. The cardiovascular function can be demonstrated through heart rate variability (HRV) measurement, including time- and frequency-domain methods^10,11^. Time-domain HRV measurement encompasses the standard deviation of all NN intervals (SDNN), the standard deviation of the averages of NN intervals in all 5-min segments of the entire recording (SDANN), the standard deviation of differences between adjacent NN intervals (SDSD), the square root of the mean of the sum of the squares of differences between adjacent NN intervals (RMSSD), the number of pairs of adjacent NN intervals differing by more than 50 ms in the entire recording (NN50 count), and the NN50 count divided by the total number of all NN intervals (pNN50)^10,11^. Frequency-domain HRV measurement, calculated based on distinct frequency components, includes total power, power in the very low range (VLF), power in the high frequency range (HF)—indicating parasympathetic and partly sympathetic activity, power in the low frequency range (LF)—indicating sympathetic activity, and the ratio of LF to HF in ms^2^ (LF/HF ratio)—indicating sympathetic or parasympathetic dominance^10,11^. Furthermore, time-domain HRV parameters including, SDSD, RMSSD, NN50 count and pNN50 can be used to estimate HF^10,11^.

Regarding the effect of green tea on HRV, a previous study showed that while green tea didn’t affect HRV in Wistar rats, it restored the reduction of LF in diabetic Wistar rats^12^. Another study revealed that green tea could increase both LF and HF, with a more significant increase observed in HF, but decrease the LF/HF ratio in Wistar rat^13^. Interestingly, a previous study found that green tea extract was an effective potentiator of sympathetically mediated thermogenesis in the brown adipose tissue of male Sprague–Dawley rats, likely as a result of a synergistic interaction between caffeine, which inhibits transcellular phosphodiesterases (enzymes that break down noradrenaline (NA)-induced cAMP)^14^, and EGCG, which inhibits catechol-*O*-methyl-transferase (the enzyme that degrades NA)^15^, thereby prolonging sympathetic stimulation^16^.

EGCG has been proposed to be involved in processes primarily regulated by the sympathetic nervous system, such as thermogenesis and fat oxidation^17^. A previous study showed that EGCG increased the mRNA expressions of *uncoupling protein 2* (*UCP2*) and *3* (*UCP3*), which are implicated in thermogenesis and energy metabolism, in the liver and skeletal muscles of obese New Zealand black mice^18^. Additionally, EGCG stimulated the expression of several enzymes in fatty acid oxidation pathways, such as medium-chain acyl-CoA dehydrogenase, in high fat-fed mice^19^. Collectively, EGCG has been shown to decrease BP and potentially enhance sympathetic activation; however, the effects of EGCG on the ANS, as reflected by HRV, have not been studied in humans. This study aimed to investigate EGCG’s effects on SBP, DBP, and mean arterial pressure (MAP) and HRV in obese subjects and determine the associations of SBP, DBP, and MAP with metabolic factors and HRV. This could shed light on the effect of EGCG on the ANS and cardiovascular function, potentially leading to the development of more effective strategies for managing ANS- and BP-related cardiovascular conditions.

## Results

### Clinical characteristics of the subjects

Clinical characteristics of the subjects at baseline, week 4, and week 8 are presented in Table 1. Age, body weight (BW), body mass index (BMI), fat percentage (% fat), and fat mass were not different between the placebo and EGCG-treated groups at baseline, week 4, and week 8, even after adjusting for age, gender, or BMI (Table 1). The gender ratio was 4:11 in the placebo group and 9:6 in the EGCG group, indicating a noticeable difference between the groups (Table 1). Interestingly, in the EGCG group, SBP, DBP, and MAP were significantly lower at week 8 compared to baseline, and DBP and MAP were significantly lower at week 8 compared to week 4 (P < 0.05 all) (Table 1). SBP, DBP, and MAP were not significantly different between the placebo and EGCG groups at baseline, week 4, and week 8, even after adjusting for age, gender, or BMI (Table 1). Remarkably, when adjusted for the baseline values of each group, SBP and MAP, but not DBP, were significantly lower in the EGCG group compared to the placebo group at week 8 (P < 0.05 all) (Table 1).

**Table 1:** Clinical characteristics of the subjects.

Variables (Mean ± SEM)	Placebo (n = 15)			EGCG (n = 15)		
	Baseline	Week 4	Week 8	Baseline	Week 4	Week 8
Age (years)	36.27 ± 2.39			35.80 ± 1.97		
Gender (M:F)	4:11			9:6		
BW (kg)	76.43 ± 3.65	76.79 ± 3.64	76.58 ± 3.72	82.51 ± 4.12	82.52 ± 4.05	82.53 ± 4.05
BMI (kg/m^2^)	29.38 ± 0.94	29.53 ± 0.94	29.39 ± 0.95	30.55 ± 1.43	30.57 ± 1.41	30.56 ± 1.42
% Fat (%)	37.51 ± 1.92	37.75 ± 1.96	36.95 ± 1.96	35.45 ± 2.40	35.84 ± 2.27	36.41 ± 2.62
Fat Mass (kg)	28.94 ± 2.40	29.07 ± 2.37	28.57 ± 2.38	29.95 ± 3.19	30.05 ± 3.23	30.84 ± 3.55
SBP (mmHg)	120.93 ± 3.94	124.20 ± 5.23	124.00 ± 5.54	122.77 ± 2.46	117.08 ± 2.45	115.85 ± 1.99**^,a^
DBP (mmHg)	81.87 ± 3.09	84.00 ± 3.12	81.73 ± 2.99	82.62 ± 1.70	81.54 ± 2.11	77.85 ± 1.79^a,b^
MAP (mmHg)	94.89 ± 3.26	97.40 ± 3.74	95.82 ± 3.81	96.00 ± 1.64	93.38 ± 1.98	90.51 ± 1.64*^,aa,b^

Demographic and anthropometric data, as well as SBP and DBP in terms of mean ± SEM for the placebo group, were already presented in a table format in our previous study^47^ under a Creative Commons license, which allows for use, sharing, adaptation, distribution, and reproduction with appropriate citation.

EGCG, Epigallocatechin gallate; M, male; F, female; BW, body weight; BMI, body mass index; % Fat, fat percentage; SBP, systolic blood pressure; DBP, diastolic blood pressure; MAP, mean arterial pressure. Data are shown as mean ± standard error of the mean (SEM); *P < 0.05, **P < 0.01 compared to the placebo group adjusted for baseline, ^a^P < 0.05, ^aa^P < 0.01 compared to baseline within the same group, ^b^P < 0.05 compared to week 4 within the same group.

### The effect of EGCG treatment on HRV

**Fig. 1:**
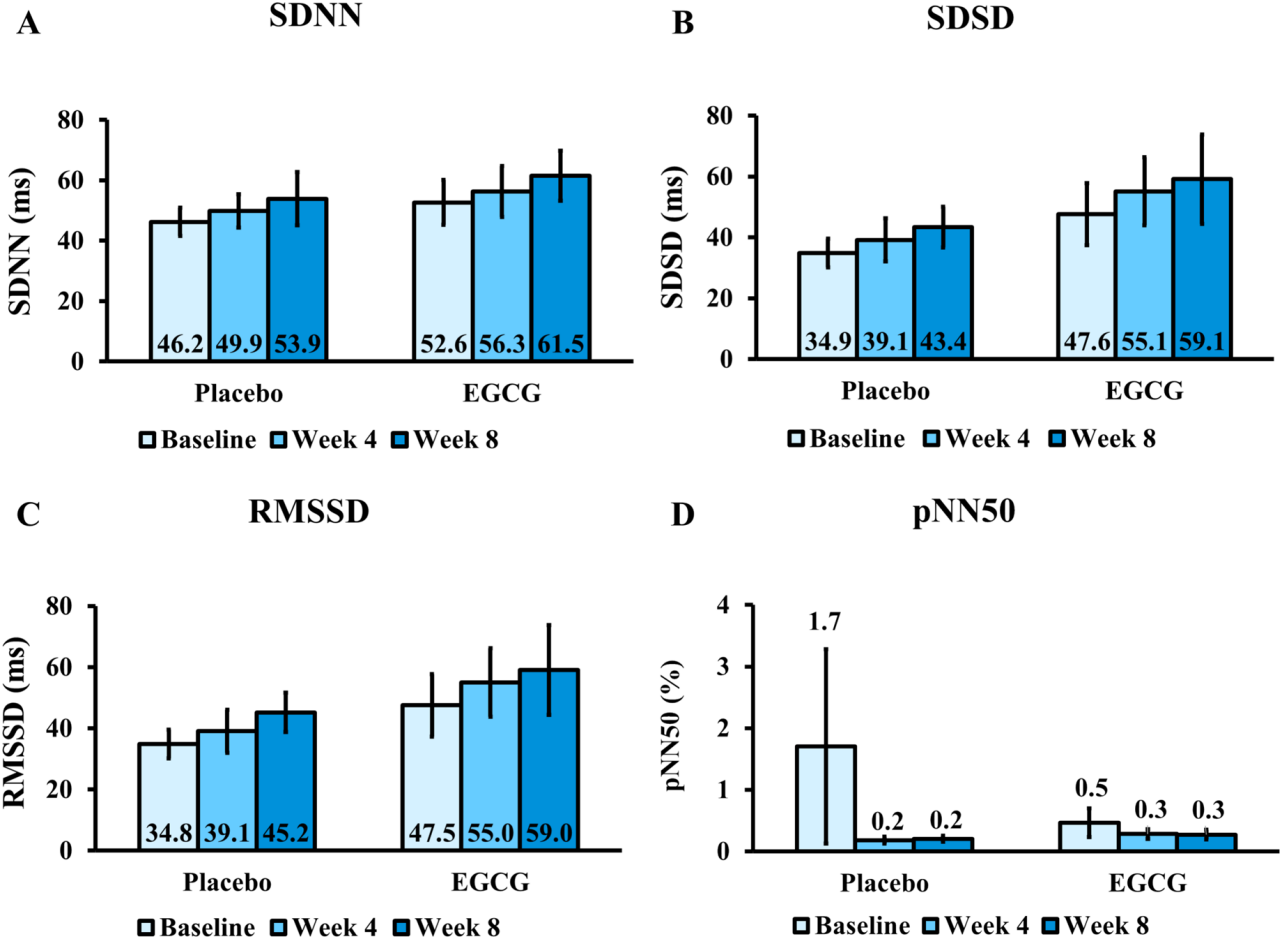
The effect of EGCG treatment on time-domain heart rate variability. EGCG, Epigallocatechin gallate; SDNN, standard deviation of all NN intervals; SDSD, standard deviation of differences between adjacent NN intervals; RMSSD, the square root of the mean of the sum of the squares of differences between adjacent NN intervals; pNN50, percent of pNN50 count.

**Fig. 2:**
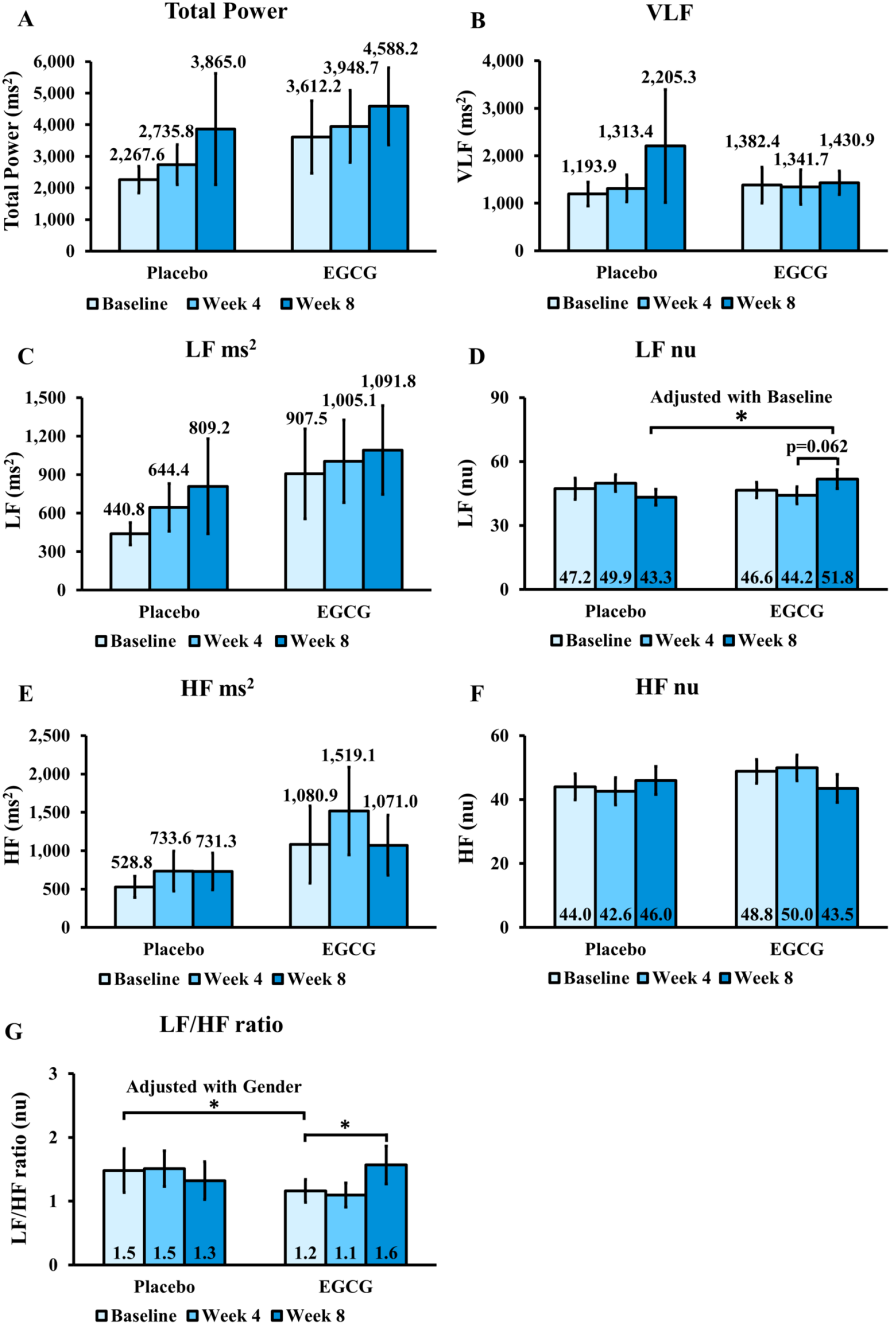
The effect of EGCG treatment on frequency-domain heart rate variability. EGCG, Epigallocatechin gallate, VLF, power in the very low frequency range; LF ms^2^, power in the low frequency range in ms^2^; LF nu, power in the low frequency range in normalized units; HF ms^2^, power in the high frequency range in ms^2^; HF nu, power in the high frequency range in normalized units; LF/HF ratio, the ratio of LF ms^2^ to HF ms^2^, *P < 0.05.

The effect of EGCG treatment on HRV is shown in Figs. 1 and 2. Time-domain HRV, including SDNN (Fig. 1A), SDSD (Fig. 1B), RMSSD (Fig. 1C), and pNN50 (Fig. 1D), and frequency-domain HRV parameters, including total power (Fig. 2A), VLF (Fig. 2B), LF in ms^2^ (LF ms^2^) (Fig. 2C), LF in normalized units (LF nu) (Fig. 2D), HF in ms^2^ (HF ms^2^) (Fig. 2E), HF in normalized units (HF nu) (Fig. 2F), and LF/HF ratio (Fig. 2G), were not significantly different between the placebo and the EGCG groups at baseline, week 4, and week 8. After adjusting for the baseline values within each group, LF nu at week 8 was significantly higher in the EGCG group compared to the placebo group (P < 0.05) (Fig. 2D). Additionally, after adjusting for gender, the LF/HF ratio was significantly lower at baseline (P < 0.05) but was comparable at week 4 and week 8 in the EGCG group compared to the placebo group (Fig. 2G). Other comparisons between the placebo and the EGCG groups remained comparable even after adjusting for age, gender, BMI, or their baseline values (Figs. 1A–D, 2A–C, E, F). Within the EGCG group, the LF/HF ratio significantly increased at week 8 compared to baseline (P < 0.05) (Fig. 2G) and LF nu tended to increase at week 8 compared to week 4 (P = 0.062) (Fig. 2D). The placebo had no effects on time-domain HRV and frequency-domain HRV (Figs. 1, 2).

### Correlations between SBP, DBP or MAP with clinical and metabolic parameters and HRV

Correlations between SBP, DBP or MAP with clinical and metabolic parameters and HRV are shown in Tables 2 and 3, respectively.

**Table 2:**
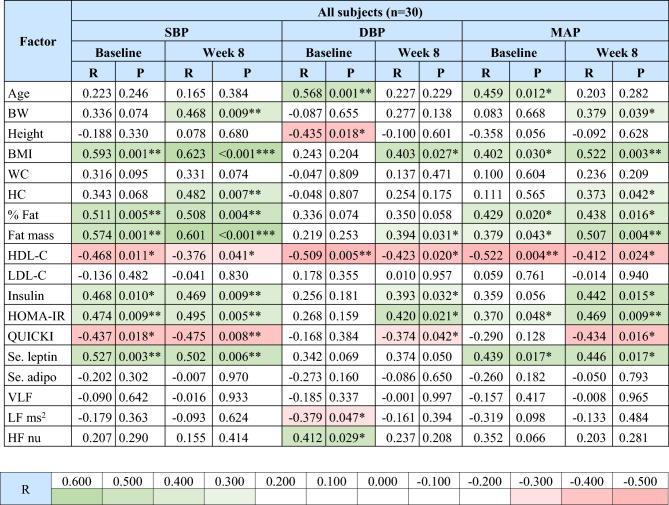
Correlations between SBP, DBP, and MAP with clinical and metabolic parameters and HRV for all subjects.

green color represents positive significant correlations,  red color represents negative significant correlations; R, correlation coefficient; SBP, systolic blood pressure; DBP, diastolic blood pressure; MAP, mean arterial pressure; BW, body weight; BMI, body mass index; WC, waist circumference; HC, hip circumference; % fat, fat percentage; HDL-C, high-density lipoprotein cholesterol; LDL-C, low-density lipoprotein cholesterol; HOMA-IR, Homeostatic Model Assessment for Insulin Resistance; QUICKI, quantitative insulin sensitivity check index; Se., serum; adipo, adiponectin; VLF, power in the very low range; LF ms^2^, power in the low frequency range in ms^2^; HF nu, power in the high frequency range in normalized units; LF/HF ratio, the ratio of LF to HF.

*P < 0.05, **P < 0.01, ***P < 0.001.

**Table 3:**
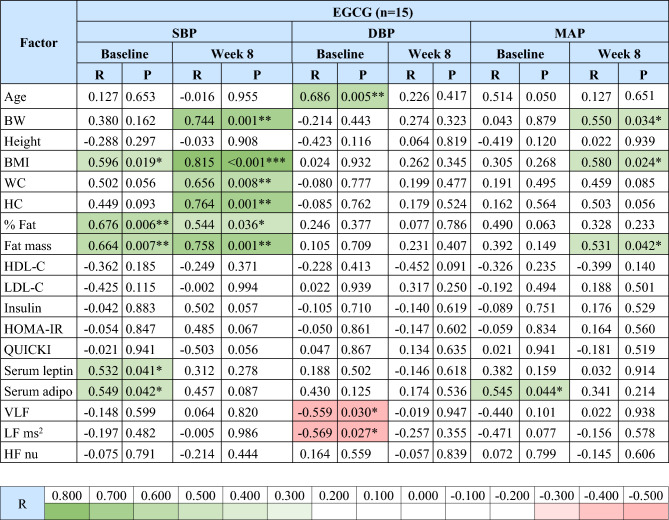
Correlations between SBP, DBP, and MAP with clinical and metabolic parameters and HRV for the EGCG group.

Green color represents significant positive correlations,  red color represents significant negative correlations; R, correlation coefficient; SBP, systolic blood pressure; DBP, diastolic blood pressure; MAP, mean arterial pressure; BW, body weight; BMI, body mass index; WC, waist circumference; HC, hip circumference; % fat, fat percentage; HDL-C, high-density lipoprotein cholesterol; LDL-C, low-density lipoprotein cholesterol; HOMA-IR, homeostatic model assessment for insulin resistance; QUICKI, quantitative insulin sensitivity check index; Se., serum; adipo, adiponectin; VLF, power in the very low range; LF ms^2^, power in the low frequency range in ms^2^; HF nu, power in the high frequency range in normalized units.

*P < 0.05, **P < 0.01, ***P < 0.001.

In all subjects, SBP had significant positive correlations with BMI, % fat, fat mass, serum insulin levels, the homeostatic model assessment for insulin resistance (HOMA-IR), and serum leptin levels at baseline and week 8; and with BW and hip circumference at week 8; but had significant negative correlations with high-density lipoprotein cholesterol (HDL-C) and the quantitative insulin sensitivity check index (QUICKI) at baseline and week 8 (P < 0.05 all) (Table 2). DBP was significantly positively correlated with age and HF nu at baseline; and with BMI, fat mass, serum insulin levels, and the HOMA-IR at week 8; but was significantly negatively correlated with HDL-C at baseline and week 8; with height and LF ms^2^ at baseline; and with the QUICKI at week 8 (P < 0.05 all) (Table 2). MAP showed significant positive correlations with BMI, % fat, fat mass, the HOMA-IR, and serum leptin levels at baseline and week 8; with age at baseline; and with BW, hip circumference, and serum insulin levels at week 8; but showed significant negative correlations with HDL-C at baseline and week 8; and with QUICKI at week 8 (P < 0.05 all) (Table 2).

In the EGCG group, SBP had significant positive correlations with BMI, % fat, and fat mass at baseline and week 8; with serum leptin and adiponectin levels at baseline; and with BW, waist circumference, and hip circumference at week 8 (P < 0.05 all) (Table 3). DBP was significantly positively correlated with age at baseline but was significantly negatively correlated with VLF and LF ms^2^ at baseline (P < 0.05 all) (Table 3). MAP showed significant positive correlations with serum adiponectin levels at baseline and with BW, BMI, and fat mass at week 8 (P < 0.05 all) (Table 3).

### Multivariate linear regression analyses of SBP, DBP and MAP for all subjects at baseline

Multivariate linear regression analyses of SBP, DBP and MAP for all subjects at baseline are shown in Table 4. When setting SBP as a dependent variable, 2 models with significant interactions were observed, using BMI (Model 1) or BMI and HF nu (Model 2) as independent variables (P < 0.05 all) (Table 4). For DBP as a dependent variable, 3 significant interactions were found, using HDL-C (Model 1), or HDL-C and height (Model 2), or HDL-C, height, and HF nu (Model 3) as independent variables (P < 0.05 all) (Table 4). Similarly, setting MAP as a dependent variable resulted in 3 significant models, using HDL-C (Model 1), or HDL-C and HF nu (Model 2), or HDL-C, HF nu, and height (Model 3), as independent variables (P < 0.05 all) (Table 4).

**Table 4:** Multiple regression analysis for SBP, DBP, and MAP for all subjects at baseline (n = 30).

Factors	R	R^2^	P value		Coeffi-cient	standard error	T value	P value
SBP model 1	0.564	0.318	0.003**	Constant	63.100	17.254	3.657	0.001**
				BMI	1.974	0.590	3.342	0.003**
SBP model 2	0.658	0.433	0.041*	Constant	51.692	16.909	3.057	0.006**
				BMI	1.932	0.550	3.512	0.002**
				HF nu	0.274	0.127	2.163	0.041*
DBP model 1	0.541	0.292	0.004**	Constant	104.611	7.547	13.861	< 0.001***
				HDL-C	− 0.505	0.161	− 3.148	0.004**
DBP model 2	0.689	0.475	0.009**	Constant	187.050	29.866	6.263	< 0.001***
				HDL-C	− 0.505	0.141	− 3.575	0.002**
				Height	− 0.505	0.178	− 2.831	0.009**
DBP model 3	0.789	0.623	0.008**	Constant	167.911	26.686	6.292	< 0.001***
				HDL-C	− 0.462	0.123	− 3.753	0.001**
				Height	− 0.474	0.155	− 3.063	0.006**
				HF nu	0.265	0.090	2.938	0.008**
MAP model 1	0.553	0.306	0.003**	Constant	118.303	7.526	15.719	< 0.001***
				HDL-C	− 0.521	0.160	− 3.255	0.003**
MAP model 2	0.678	0.459	0.018*	Constant	103.845	8.844	11.741	< 0.001***
				HDL-C	− 0.478	0.145	− 3.286	0.003**
				HF nu	0.271	0.106	2.550	0.018*
MAP model 3	0.744	0.554	0.042*	Constant	164.449	29.247	5.623	< 0.001***
				HDL-C	− 0.479	0.135	− 3.551	0.002**
				HF nu	0.257	0.099	2.594	0.017*
				Height	− 0.367	0.170	− 2.159	0.042*

R, correlation coefficient; SBP, systolic blood pressure; DBP, diastolic blood pressure; MAP, mean arterial pressure; BMI, body mass index; HDL-C, high density lipoprotein cholesterol; HF nu, HF power in normalized units. *P < 0.05, **P < 0.01, ***P < 0.001.

## Discussion

This study investigated the effects of EGCG on SBP, DBP, MAP, and HRV parameters in obese subjects, along with the correlations between BP, clinical data, metabolic profiles, and HRV parameters. Additionally, multiple linear regression analyses for SBP, DBP, and MAP were conducted. To the best of our knowledge, this is the first study exploring the impact of EGCG on HRV parameters, which reflect the ANS in humans.

Our findings indicate that an 8-week EGCG treatment significantly reduced SBP by 6.92 mmHg, DBP by 4.77 mmHg, and MAP by 5.49 mmHg from baseline in obese individuals. Furthermore, DBP and MAP of the EGCG-treated group were significantly lower at week 8 compared to week 4, with reductions of 3.69 mmHg and 2.87 mmHg, respectively. In contrast, there was no significant difference between time points in the placebo group. When adjusted for baseline values of each group, SBP and MAP at week 8 were significantly lower in the EGCG-treated group compared to the placebo group. These findings were consistent with previous studies demonstrating EGCG’ s ability to lower both SBP^4,5^ and DBP^4–6^ in obese subjects.

Clinical characteristics, as well as SBP, DBP, and MAP, were not significantly different at baseline; however, the gender ratio seemed to differ between the placebo and EGCG groups. To account for this issue, we adjusted clinical characteristics, SBP, DBP, MAP, and HRV for age, gender, or BMI. We ensured that any observed differences or comparability were not influenced by these factors, as the same statistical results were observed after these adjustments, except for the LF/HF ratio. While the LF/HF ratio was not significantly different between the placebo and EGCG groups at baseline before adjustment, it was significantly lower in the EGCG group compared to the placebo group after adjusting for gender, and this will be discussed in more detail later.

Given that the mean SBP reduced from 122.77 to 115.85 mmHg and DBP from 82.62 to 77.85 mmHg, both of which fall within the normotensive range^20^ (less than 140/90 mmHg), it is notable that 2 subjects were initially in the hypertensive range for both SBP and DBP at baseline, while only 1 subject remained in this range at week 8. Although most subjects were normotensive, the reduction in BP can be beneficial, as it may help reduce the risk of developing cardiovascular conditions in the future. According to the 2023 ESH Guidelines for the management of arterial hypertension, the optimal BP is less than 120/80 mmHg^20^. A meta-analysis from 61 prospective cohort studies found that cardiovascular events and mortality rates increased when BP rose from 115/75 mmHg, which was lower than the BP of every subject in this study^21^. There was no strong evidence of adverse effects from this BP level^21^. Additionally, our subjects are obese, increasing their risk of developing cardiovascular disease compared to the general population. Therefore, reducing BP in our obese subjects may be considered advantageous. However, healthy individuals with lower BP than this level are less likely to benefit from further reduction of BP and may experience some minor side effects such as dizziness, fatigue, and depressed mood^21,22^.

For the HRV results, LF nu at week 8 was significantly higher in the EGCG group compared to the placebo group after adjusting for baseline values within each group. In the EGCG group, but not the placebo group, LF nu tended to increase at week 8 compared to week 4. Additionally, the LF/HF ratio significantly increased at week 8 compared to baseline in the EGCG group, but not in the placebo group. For the LF/HF ratio, there was no significant difference between the placebo and EGCG groups at baseline, week 4, and week 8. However, after adjusting for gender, the LF/HF ratio in the EGCG group, which was lower at baseline, increased to become comparable to the placebo group at week 8. This indicates that the EGCG group experienced an increase in the LF/HF ratio after 8 weeks of treatment, consistent with the within-group comparisons. The placebo had no effects on time-domain or frequency-domain HRV. Collectively, there were increases in LF nu and the LF/HF ratio in the EGCG group. Increased LF nu represents elevated sympathetic activity, while an increased LF/HF ratio indicates increased sympathetic and/or decreased parasympathetic activity. Additionally, DBP exhibited a significant negative correlation with LF ms^2^ and a significant positive correlation with HF nu, suggesting that a decrease in DBP is associated with increased sympathetic and decreased parasympathetic activity. The shift toward sympathetic dominance in HRV in the EGCG-treated group might be because EGCG potentially enhances sympathetic activity through various mechanisms, including inhibiting catechol-*O*-methyltransferase as shown in rats and mice, which prolongs sympathetic activity^15^; increasing *UCP2* and *UCP3* mRNA expressions as reported in obese New Zealand black mice^18^; and stimulating the expression of several enzymes involved in fatty acid oxidation pathways through activation of Sirtuin 1 (SIRT1) and AMP-activated protein kinase (AMPK) as observed in mice^23^. Furthermore, a preliminary randomized clinical trial showed that 300 mg of EGCG decreased the postprandial respiratory quotient in obese male subjects^24^, and the EGCG/caffeine group increased fat oxidation measured by indirect calorimetry by 10.6% compared to the placebo, indicating a potential role of EGCG in increasing fatty acid oxidation^25^. Collectively, we propose that the increase in LF nu and LF/HF ratio observed after 8 weeks of EGCG treatment aligns with enhanced sympathetic activity induced by EGCG. Our study is the first to confirm that 8 weeks of EGCG treatment shifts HRV toward increased sympathetic dominance in humans.

Another explanation of a shift toward sympathetic dominance after 8 weeks of EGCG treatment might be from a compensatory response after a decline in SBP, DBP, and MAP. EGCG has the vasodilatory effect through stimulation of NO release and suppression of ET1 production^8,9^, thereby lowering BP, triggering a compensatory response to increase BP by stimulating the sympathetic nervous system and suppressing the parasympathetic system^26^.

The correlation and regression analyses of baseline values of all subjects further elucidated the relationship between BP and various metabolic factors. SBP showed positive correlations with obesity (BMI, % fat, fat mass, and serum leptin levels) and insulin resistance (insulin and HOMA-IR) factors, and a negative correlation with the insulin sensitivity parameter (QUICKI). Furthermore, regression analysis showed that BMI was the major factor contributing to SBP. These results suggest that SBP was associated with increased obesity and insulin resistance, while being inversely related to insulin sensitivity. Our study aligns with previous studies revealing increased serum leptin levels in patients with essential hypertension^27,28^. Prior studies have shown that leptin is required for an increase in BP in diet-induced obesity mice^29^, stimulates sympathetic nervous system, and inhibits NO production, leading to increased renal sodium reabsorption^28,30,31^. Moreover, high insulin levels can directly promote renal sodium reabsorption and stimulate sympathetic nervous system activity^31,32^. Taken together, the elevated systolic SBP in obese individuals is, at least in part, attributed to stimulated sympathetic nervous system activity and enhanced renal sodium reabsorption, mediated by increased levels of leptin and insulin.

For the correlation analysis of SBP in the EGCG group, the power to detect significant correlations was reduced due to the smaller sample size (n = 15). Interestingly, after 8 weeks of EGCG treatment, more significant correlations were observed with obesity parameters such as BW, BMI, waist circumference, hip circumference, % fat, and fat mass, while fewer significant correlations were noted with serum leptin and adiponectin levels, though the direction of these correlations remained consistent with pre-treatment.

For DBP, it revealed a significant positive correlation with age, which could be explained by the stiffening of peripheral blood vessels with aging leading to increased peripheral vascular resistance, and consequently, DBP^33^. Furthermore, DBP exhibited a negative correlation with height and regression analysis showed that height was the major negative contributor to DBP. A previous study has demonstrated a negative correlation between DBP and height in non-obese subjects^34^. This is attributed to the fact that shorter individuals may experience an early reflected arterial wave, leading to increased left ventricular afterload and arterial stiffness. In some cases, however, no correlation was found^35^. This discrepancy might be because other studies were conducted in individuals who were not obese, whereas our study focused on obese individuals, which might exhibit changes in metabolic factors that could influence these results. Correlation analysis revealed that DBP had a positive correlation with HF nu and a negative correlation with LF ms^2^. Furthermore, multiple linear regression analyses revealed that HF nu provided a significant positive contribution to SBP and DBP, indicating that reductions in SBP and DBP were associated with a reduction in parasympathetic activity. Previous studies showed that DBP was negatively correlated with HF and LF, while SBP was positively correlated with LF but negatively with HF in young adults with a cardiovascular risk^36^. Additionally, LF and HF are lower in hypertensive subjects^37^. A negative correlation between DBP and LF aligns with prior studies^36,37^, but this is not consistent for HF^36,37^. This inconsistency observed in our study may be explained by autonomic imbalance in obese individuals^38,39^.

For the correlation analysis of DBP in the EGCG group after 8 weeks of treatment, the positive correlation with age and the negative correlations with VLF and LF ms^2^ observed at baseline disappeared. The shift from a significant positive correlation to no significant correlation between DBP and age suggests that EGCG may reduce an age-related increase in DBP. Additionally, the disappearance of the negative correlation between DBP and VLF and LF from baseline indicates that EGCG may alter the sympathetic and parasympathetic balance influencing DBP.

Correlation analyses of MAP showed consistent correlations with SBP, including BMI, % fat, fat mass, HOMA-IR, and serum leptin levels, and with DBP, including age, in our study. Moreover, regression analysis of MAP revealed that height was a significant contributor to MAP, similar to the regression analysis of DBP. HF nu and LF ms^2^ had a trend of negative and positive correlation with MAP, respectively, but it wasn’t significant since MAP is a combination of SBP and DBP, and SBP wasn’t correlated with these factors.

For the correlation analysis of MAP in the EGCG group, positive correlations with BW, BMI, and fat mass increased from non-significant to statistically significant from before to after 8 weeks of treatment, while the correlation with serum adiponectin levels decreased from significant to non-significant, indicating a shift towards stronger correlations with obesity parameters and weaker correlations with hormones involved in insulin sensitivity after treatment, similar to SBP.

**Fig. 3:**
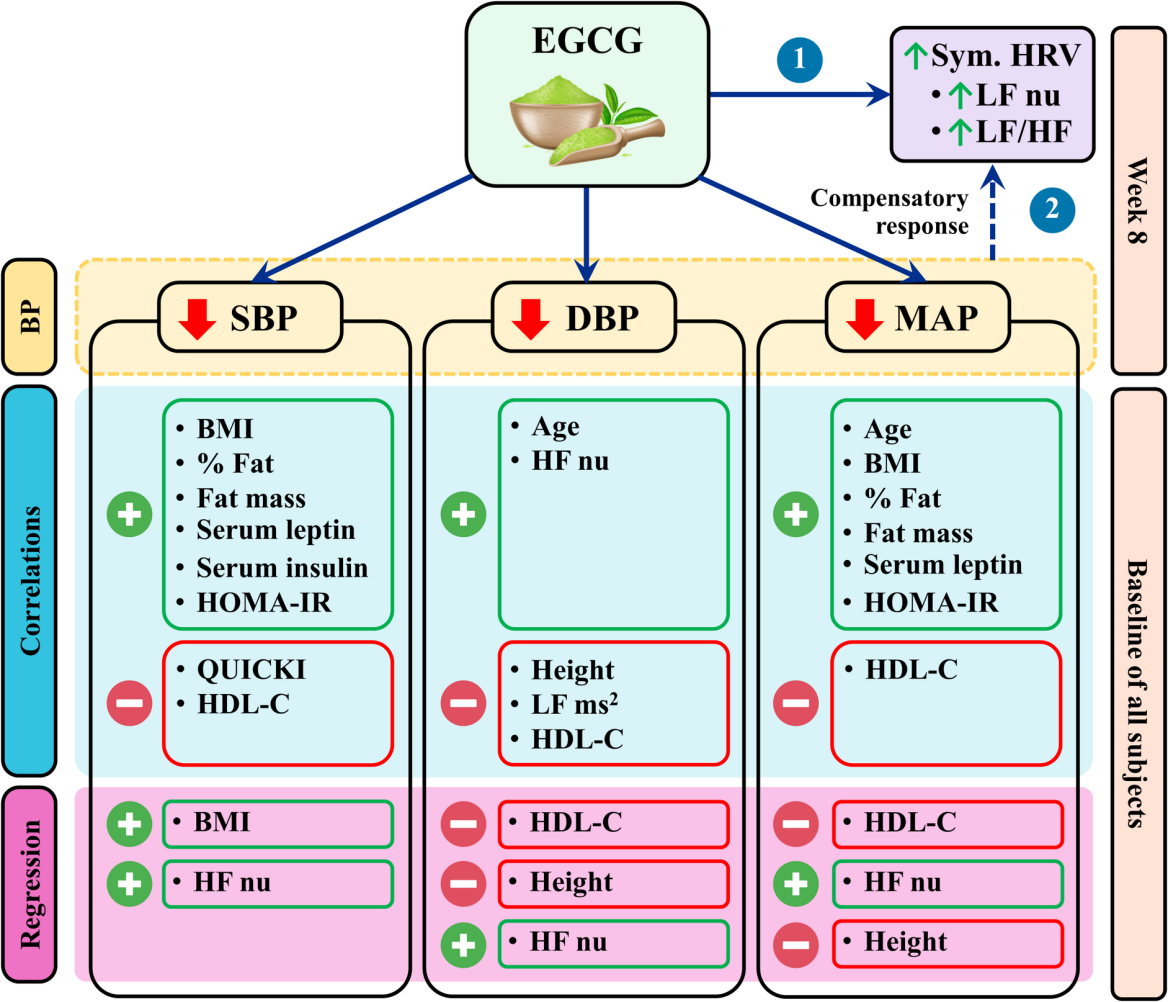
The summary of results. EGCG, Epigallocatechin gallate; BP, blood pressure; SBP, systolic blood pressure; DBP, diastolic blood pressure; MAP, mean arterial pressure; Sym., sympathetic; HRV, heart rate variability; LF nu, power in the low frequency range in normalized units; LF/HF ratio, the ratio of LF ms^2^ to HF ms^2^; BMI, body mass index; % fat, fat percentage; HOMA-IR, Homeostatic Model Assessment for Insulin Resistance; HF nu, power in the high frequency range in normalized units; QUICKI, quantitative insulin sensitivity check index; HDL-C, high density lipoprotein cholesterol, LF power in ms^2^, power in the low frequency range in ms^2^, dotted arrows represent proposed mechanism, , positive correlations and regressions; , negative correlations and regressions; , first mechanism; , second mechanism.

Interestingly, for all subjects at baseline, HDL-C exhibited negative correlations with SBP, DBP, and MAP, and in regression analyses, it was a main negative contributor to DBP and MAP, reflecting the protective role of HDL in the cardiovascular system. These findings correlate with a previous study that showed negative correlations of SBP and DBP with HDL-C in humans^40^. HDL-C regulates blood cholesterol levels by the reverse cholesterol transport mechanism, which transports cholesterol to the liver, where it undergoes a transformation into bile acid or is directly excreted into bile^41^. Furthermore, HDL-C has anti-inflammatory and anti-oxidative properties which downregulate several pro-inflammatory molecules, upregulate nitric oxide, and prevent lipid oxidation, thereby potentially improving endothelial function and leading to lower SBP, DBP, and MAP^41^. The summary of results is shown in Fig. 3.

This study encountered some limitations. Firstly, as our primary focus was to assess the impact of the EGCG supplement, both energy consumption and expenditure were not tightly controlled; instead, food records were employed to monitor any significant changes in energy consumption. Secondly, although we accounted for dietary sources of EGCG, such as tea and coffee, through detailed questionnaires and logbooks, there may still be residual confounding from other sources of EGCG not fully captured in our data. Thirdly, a 5-min (short term) HRV measurement might not be the gold standard of HRV measurement compared to 24-h HRV; nevertheless, it’s applicable for such short-time experiments^11^. Fourthly, since EGCG might affect the ANS, we have excluded subjects using drugs and those with diseases that can affect the autonomic nervous system, such as diabetes mellitus; however, we did not exclude subjects with conditions such as hypertension and neurological diseases^42^, which could potentially influence the outcomes related to HRV and BP regulation, as these conditions might alter autonomic responses. Future studies should consider excluding such subjects to better isolate the effects of EGCG on the measured outcomes.

## Conclusion

Our study shows that 8 weeks of EGCG treatment significantly reduced SBP, DBP, and MAP but elevated LF nu and LF/HF ratio, indicating a shift towards sympathetic nervous system dominance and/or decreased parasympathetic nervous system activity. These results might stem from the direct effect of EGCG as a sympathetic potentiator or a compensatory response after its primary vasodilatory effect in reducing BP. For all subjects at baseline, SBP was associated with obesity and insulin resistance factors; DBP was positively associated with HF nu and negatively correlated with LF ms^2^; and MAP was correlated with obesity and insulin resistance parameters, consistent with SBP. After 8-week EGCG treatment, the correlation coefficients of SBP, DBP, and MAP with other factors changed, being mostly more correlated with adiposity but less with serum leptin and adiponectin levels for SBP and MAP, and less correlated with age and HRV parameters for DBP. Additionally, the negative correlations between HDL-C and SBP, DBP, and MAP underscores its potential protective role against hypertension. These insights offer a foundational understanding of the HRV changes post-EGCG treatment, potentially making it the first study in humans to show that EGCG stimulates sympathetic activity. Further research is needed to elucidate the potential mechanisms of EGCG in enhancing sympathetic activity in humans, which could lead to better management of the ANS and cardiovascular health.

## Materials and methods

### Ethics statement

The human study was approved by the Siriraj Institutional Review Board of the Faculty of Medicine Siriraj Hospital, Mahidol University, (COA. Si 563/2015) according to international guidelines for human research protection, such as the Declaration of Helsinki, the Belmont Report, CIOMS Guidelines, and the International Conference on Harmonization in Good Clinical Practice (ICH-GCP). Additionally, the study’s protocol was registered with the Thai Clinical Trials Registry (TCTR) on 21/04/2020 at 20:31:49, under the identification number TCTR20200422001, which can be accessed via https://www.thaiclinicaltrials.org/show/TCTR20200422001. Prior to the study, all participants provided signed informed consent.

### The study protocols

This study is a double-blind, randomized controlled trial in humans, performed at the Department of Physiology, Faculty of Medicine Siriraj Hospital from October 2015 to May 2019. The 30 subjects were randomly allocated into the EGCG group (n = 15) and the placebo group (n = 15), which received 150 mg of EGCG and a starch capsule, respectively, twice daily after breakfast and dinner without any dietary restriction for 8 weeks. The 95% purified EGCG, produced by Shaanxi Jiahe Phytochem Co., Ltd, Ki’an, China (Lot. No STP-QCP-133-091), was encapsulated by Bangkok lab & cosmetic Co., Ltd, Ratchaburi, Thailand. All supplement capsules were identical in shape and appearance, and the type of supplement each subject received was not disclosed to both participants and researchers. The random allocation was done using 30 drawing cards (15 per group), performed by the researchers, placed in a bottle, and picked by the subjects themselves. Clinical and anthropometric data, along with spontaneous 5-min HRV measurements, were collected 3 times at baseline, week 4 and week 8 of the experiment. The 8-h fasting blood samples were collected twice at baseline and week 8, to measure metabolic and hormonal levels. Throughout the entire study, each subject was instructed to maintain their normal physical activity and diet. Participants were instructed to maintain their normal physical activity and diet throughout the study. Additionally, they were provided with logbooks containing calendars and a questionnaire to track their diets for 3 days before each data collection point, mark supplement intake days, provide information on tea and coffee consumption, and report any side effects. To ensure adherence, subjects were required to bring their logbooks and remaining supplements at the end of weeks 4 and 8. The safety and adverse effects of the 8-week EGCG treatment are addressed in our previously published article^4^.

### Subjects

**Fig. 4:**
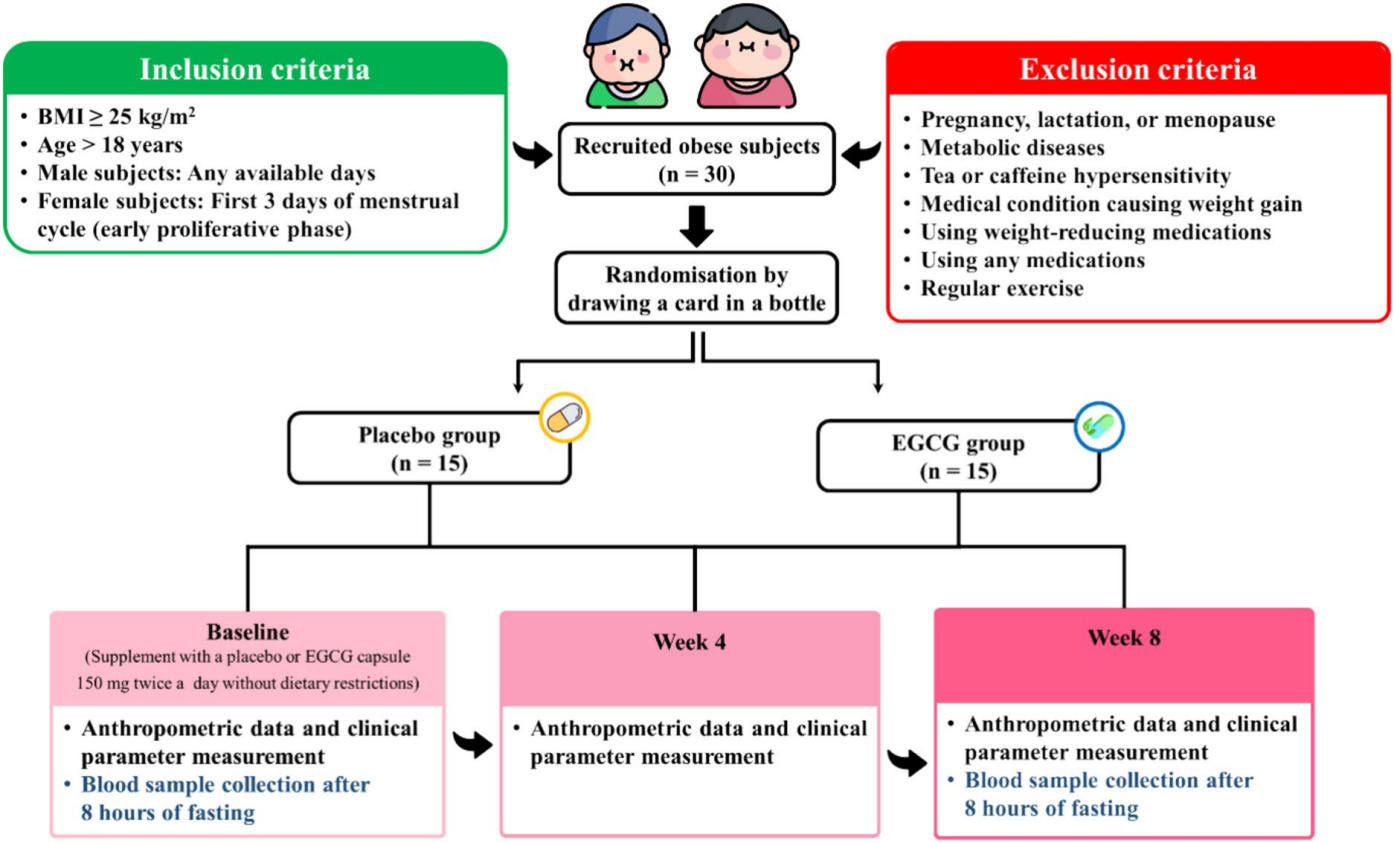
Allocation of subjects. The figure was adapted from a previous study^47^ under a Creative Commons license, which allows for use, sharing, adaptation, distribution, and reproduction with appropriate citation. BMI, body mass index; EGCG, Epigallocatechin gallate.

Inclusion criteria for this study were Thai obese subjects aged over 18 years with a BMI ≥ 25 kg/m^2^ (criteria for an Asian population)^43,44^ which is different from BMI classification of Caucasian population^45^. Male subjects were eligible to participate in the study on any available day. Female subjects, however, were required to participate during days 1–3 of their menstrual cycle, the early proliferative phase, to minimize the confounding effects of sex hormones. Exclusion criteria involved subjects with metabolic diseases such as Cushing syndrome, diabetes mellitus, thyroid diseases, and diseases causing secondary obesity; a history of hypersensitivity to tea or caffeine; use of medication for weight loss; regular exercise (defined as at least three sessions per week, each lasting 30 min^46^; and conditions such as menopause, pregnancy, and lactation. All enrolled subjects successfully completed the course of the study. The figure illustrating the allocation of subjects, adapted from a previous study^47^ under a Creative Commons license, which allows for use, sharing, adaptation, distribution, and reproduction with appropriate citation, is shown in Fig. 4.

### Demographic and anthropometric data and BP measurement

Demographic and anthropometric data, as well as SBP and DBP in terms of mean ± standard error of the mean (SEM) for the placebo group, were already presented in a table format in our previous study^47^. Since the study is under a Creative Commons license, we also present this information in Table 1. Furthermore, demographic and anthropometric data, as well as SBP and DBP in terms of the 25th percentile, median, 75th percentile, and boxplot of the EGCG group, were published in our previous study^4^. Now, we present this information in terms of mean ± SEM in Table 1. Waist circumference was measured at the umbilical level in the upright position during quiet breathing^48^ and hip circumference was determined at the broadest area of the buttocks^49^. Body weight, % fat, and fat mass were assessed by TANITA®. SBP and DBP were measured in the supine position using a sphygmomanometer after 30 min of bed rest.

### HRV measurement

The HRV measurements were performed in a silent room maintained at a temperature of 23–25 °C and a humidity level of 50–60%, following a 12-h fasting period. The subjects rested in bed for 15 min before undergoing a 15-min electrocardiogram (ECG) recording in the supine position. The recording was performed using the Finometer® Pro device, equipped with an ECG module (lead II), and was analyzed using the BeatScope® Easy program (Finapres Medical System BV, Amsterdam, The Netherlands), at a sampling rate of 200 Hz. A 5-min segment of the stable ECG recording, without ectopic beats, was selected to derive the time- and frequency-domain HRV parameters using LabChart®8Pro software (ADInstruments, Castle Hill, Australia), for which we hold a license to utilize its full range of capabilities in recording, analyzing, and visualizing ECG data. Time-domain parameters include: NN interval, which is the interval between normal, SDNN, SDANN, RMSSD, SDSD, NN50, and pNN50^10^. Frequency-domain parameters include: total power which indicates the variance of NN intervals over the temporal segment, VLF, LF ms^2^, LF nu which calculates by LF ms^2^/(total power − VLF) × 100, HF ms^2^, HF nu which is calculated by HF ms^2^/(total power − VLF) × 100, and the ratio of LF ms^2^ to HF ms^2^ (LF/HF ratio)^10^.

### Metabolic assay

The 8-h fasting insulin and HDL-C levels were examined by the central laboratory at the Department of Clinical Pathology, Faculty of Medicine Siriraj Hospital, Mahidol University, Thailand, as shown in our previous publication^4^. The HOMA-IR was determined using the equation: HOMA-IR = (fasting glucose (mg/dL) × fasting insulin (µU/mL))/405 and the QUICKI was computed with the formula: QUICKI = 1/((log(fasting insulin µU/mL) + log(fasting glucose mg/dL))^50^.

### Analysis of serum leptin levels

Serum leptin levels were determined by a commercial enzyme-linked immunosorbent assay. The detection range of leptin was 0.313–20 ng/mL, with a minimum detectable concentration of 0.313 ng/mL. For leptin analysis, serum samples were diluted in a five-fold dilution with assay buffer. The intra-assay variation of leptin was 6.66%, while the inter-assay variation was 6.74%. The optical density (O.D.) readings were taken at a wavelength of 450 nm using the Synergy HT Multi-Detection Microplate Reader, manufactured by BioTek Instruments, Inc., located in Winooski, VT, U.S.

### Statistical analysis

Statistical analysis was conducted using Statistics Package for Social Sciences (SPSS) software version 18 (IBM Corporation, New York, USA). The test of normality was performed using the Kolmogorov–Smirnov test. Comparisons between the placebo and EGCG-treated groups at baseline, week 4, and week 8 were performed using a 2-way analysis of variance (ANOVA). Comparisons between baseline, week 4, and week 8 in each group were calculated by repeated measures ANOVA followed by Fisher’s Least Significant Difference (LSD) analysis where appropriate. Additionally, the analysis of covariance (ANCOVA) was performed to adjust for age, gender, or BMI, ensuring any observed differences or comparability were not influenced by these factors, and was also used at weeks 4 and 8 to adjust for baseline values for each treatment. Given that significant interactions were found, post hoc or pairwise comparisons were conducted to further investigate which specific groups and time points exhibited significant differences. Correlations between two factors were determined using Pearson correlation for normally distributed data or Spearman correlation for non-normally distributed data. The correlation analysis was performed at baseline and week 8 for all subjects (n = 30), which requires an r-value of 0.361 to reach statistical significance, and separately for the EGCG group (n = 15), which needs an r-value of 0.515 to achieve statistical significance. The smaller sample size in the EGCG group reduced the power to detect significant correlations; however, the analysis was still performed to provide valuable insights into changes after 8 weeks of EGCG treatment. Multiple regression analysis was used to determine which factors mainly contributed to SBP, DBP, and MAP and was conducted only at baseline, pooling data from the placebo and EGCG groups, as their clinical characteristics at baseline were not different. The small sample size made separate analyses impractical, and combining data at weeks 4 and 8 from two groups was not inappropriate. A P value of 0.05 indicates statistical significance.

## Data Availability

The data that support the findings of this study are available from the corresponding author, upon reasonable request.
